# Predicting depression by using a novel deep learning model and video-audio-text multimodal data

**DOI:** 10.3389/fpsyt.2025.1602650

**Published:** 2025-09-24

**Authors:** Yifu Li, Xueping Yang, Meng Zhao, Jiangtao Wang, Yudong Yao, Wei Qian, Shouliang Qi

**Affiliations:** ^1^ College of Medicine and Biological Information Engineering, Northeastern University, Shenyang, China; ^2^ Key Laboratory of Intelligent Computing in Medical Image, Ministry of Education, Northeastern University, Shenyang, China; ^3^ Department of Psychology, The People’s Hospital of Liaoning Province, Shenyang, China; ^4^ Department of Electrical and Computer Engineering, Stevens Institute of Technology, Hoboken, NJ, United States

**Keywords:** deep learning, depression, multimedia, information fusion, local and global features

## Abstract

**Objective:**

Depression is a prevalent mental health disorder affecting millions of people. Traditional diagnostic methods primarily rely on self-reported questionnaires and clinical interviews, which can be subjective and vary significantly between individuals. This paper introduces the Integrative Multimodal Depression Detection Network (IMDD-Net), a novel deep-learning framework designed to enhance the accuracy of depression evaluation by leveraging both local and global features from video, audio, and text cues.

**Methods:**

The IMDD-Net integrates these multimodal data streams using the Kronecker product for multimodal fusion, facilitating deep interactions between modalities. Within the audio modality, Mel Frequency Cepstrum Coefficient (MFCC) and extended Geneva Minimalistic Acoustic Parameter Set (eGeMAPS) features capture local and global acoustic properties, respectively. For video data, the TimeSformer network extracts both fine-grained and broad temporal features, while the text modality utilizes a pre-trained BERT model to obtain comprehensive contextual information. The IMDD-Net’s architecture effectively combines these diverse data types to provide a holistic analysis of depressive symptoms.

**Results:**

Experimental results on the AVEC 2014 dataset demonstrate that the IMDD-Net achieves state-of-the-art performance in predicting Beck Depression Inventory-II (BDI-II) scores, with a Root Mean Square Error (RMSE) of 7.55 and a Mean Absolute Error (MAE) of 5.75. A classification to identify potential depression subjects can achieve an accuracy of 0.79.

**Conclusion:**

These results underscore the robustness and precision of the IMDD-Net, highlighting the importance of integrating local and global features across multiple modalities for accurate depression prediction.

## 1 Introduction

Depression is the primary mental health disorder contributing to the disease burden and it impacts roughly 300 million individuals worldwide (1). Depression is a widespread, costly, and debilitating condition that significantly increases the risk of suicide (2). More than 80% of individuals fail to receive appropriate treatment due to the lack of early intervention services and treatments for depression and researchers estimate that approximately one in five individuals will experience depression at some point in their lifetime (3). Consequently, it is evident that the diagnosis and screening of depression are essential.

Current approaches to evaluating depression rely predominantly on the verbal accounts provided by patients, their families, or caregivers, whether through clinical interviews or questionnaires (4). However, these traditional methods have certain limitations because the subjectivity of individuals can affect responses to questions, and symptoms of depression may manifest differently across individuals (5). Traditionally, accurate diagnosis of depression severity requires comprehensive information and extensive clinical training (6). Fortunately, advanced computing methods, such as machine learning, deep learning, and artificial intelligence, are ideally suited to enhance the assessment of mental health outcomes for individuals (7).

Utilizing audio and video methods for detecting depression offers distinct advantages, including the ability to capture direct cues and subtle behavioral changes that may not be evident in traditional assessments, while numerous indicators are used to detect depression, such as hormonal imbalances (8), changes in sleep patterns (9), cognitive performance assessments (10), resting-state functional magnetic resonance imaging (fMRI) data (11), EEG data (12, 13) and other physiological data (14). In recent years, a variety of automatic depression estimation (ADE) systems have emerged (7, 15, 16). These systems automatically assess the severity of depression using audiovisual cues, employing advanced techniques from machine learning and deep learning (17). Research indicates that speech exhibits numerous unique characteristics that can be used to identify an individual’s mental state (18–20). With the aid of various gestures involving the eyes, mouth, nose, and hands, emotions such as anger, happiness, sadness, and neutrality can be identified through depression detection systems that utilize image and video processing (21). Similarly, textual information can also be analyzed to extract features relevant to depression (22) Similarly, textual information can also be analyzed to extract features relevant to depression (22, 23). And many researchers also have explored diagnosing depression through social networks by textual information (24–26). While unimodal approaches can be effective to some extent in detecting depression, multimodal or hybrid modalities often exhibit superior performance. However, the selection of different modalities, the choice of network architectures, and the methods of fusion all significantly impact the effectiveness of depression detection (17). Many depression detection networks utilize multimodal architectures, yet few of these systems effectively incorporate both local and global features within various modalities.

**Figure 1:**
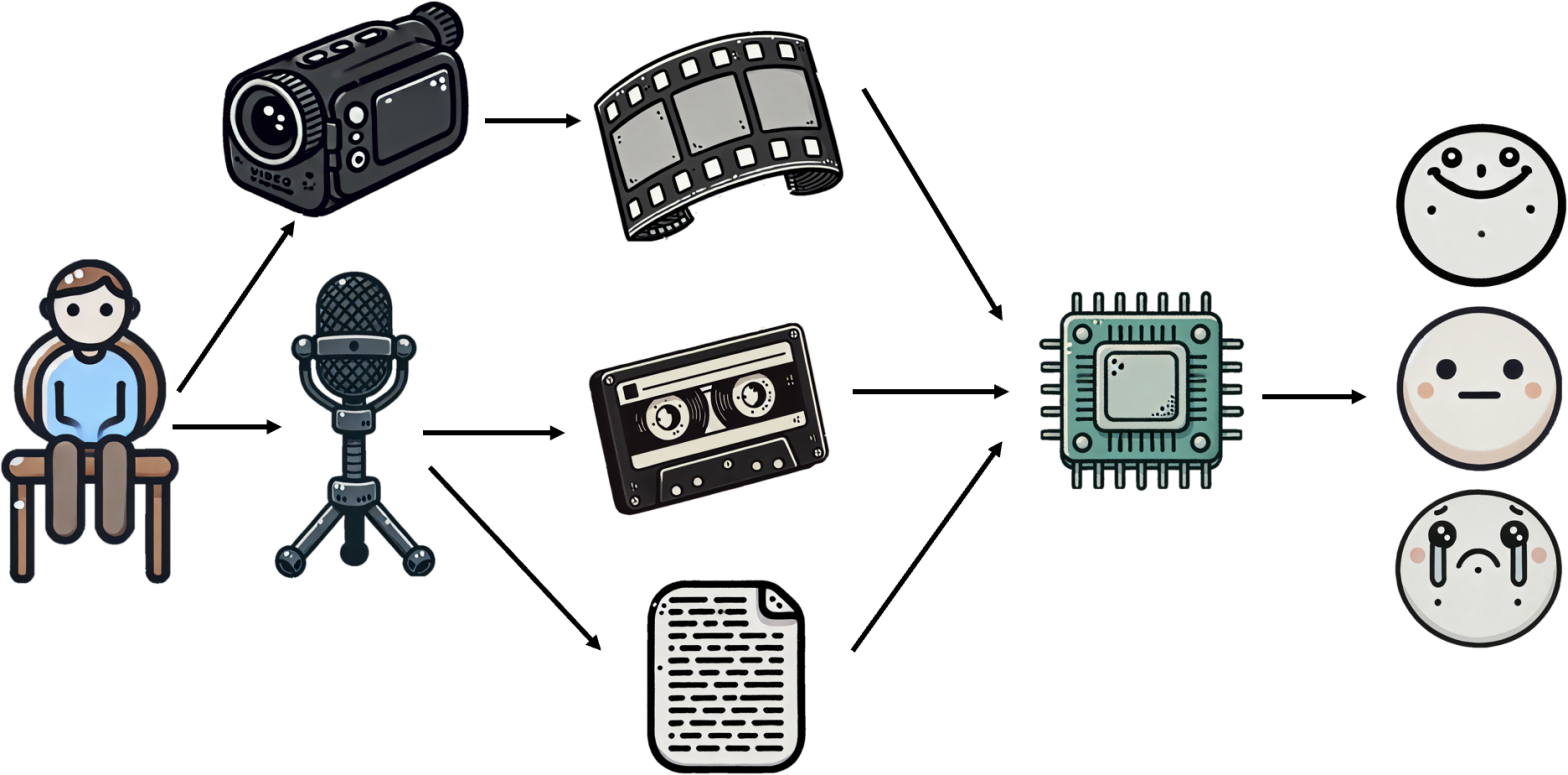
Brief diagram of multimodal depression detection by IMDD.

Consequently, this study introduces a novel deep learning network architecture for multimodal depression detection (**Figure 1**), termed the Integrative Multimodal Depression Detection Network (IMDD-Net). This advanced framework not only integrates data from video, text, and audio modalities but also considers both local and global information within each modality. By doing so, the IMDD-Net enhances the estimation efficacy by capturing a more comprehensive representation of depressive symptoms. Specifically, within the audio modality, we preprocessed the audio signals and extracted both time-frame level and global statistical features. Both sets of features were then fed into the IMDD-Net through separate channels. For video data, we sampled the video at regular intervals and processed each frame. Then we utilized the specific network to extract both global and local features. In the text modality, we employed a pre-trained Bert-base-german-cased (27) network. The high-dimensional features obtained from these three modalities were fused. This IMDD-Net was evaluated by using the AVEC2014 dataset (28), demonstrating the effectiveness of our approach in the practical assessment of depression.

The primary innovations and contributions of this paper are summarized as follows:

1. A novel multimodal network architecture has been proposed for the identification of depression;
2. This network effectively integrates and analyzes both global and local features across multiple modalities, including text, video, and audio;
3. Using the Kronecker product for multimodal fusion explores deep interactions between modalities and enhances the detection accuracy of depression.
4. The IMDD-Net achieves state-of-the-art performance in depression assessment areas.

The remainder of this paper is organized as follows. Section 2 reviews the related work on automatic depression estimation. Section 3 describes the dataset used in this study, details the data preparation and preprocessing steps, and provides a comprehensive explanation of the proposed IMDD-Net architecture. Section 4 presents the experimental results obtained using IMDD-Net and provides an analysis of the model’s performance across regression and classification tasks. Section 5 discusses the implications of our findings, outlines current limitations, and considers directions for future improvement and clinical applicability. Section 6 concludes the paper and summarizes the main contributions of this work.

## 2 Related works

Recent years have witnessed significant advancements in the field of automatic depression estimation through deep learning. In 2021, Dong et al. (19) developed an automatic depression estimation method using speech signals. This approach combines deep speaker recognition and speech emotion recognition features from pre-trained models to utilize complementary vocal and emotional data. In 2020, Li et al. (29) introduced DRR_DepressionNet to predict depression severity from facial expressions. This method enhances facial images to enlarge the training dataset and uses a modified ResNet divided into C_M block, Resblock, and global average pooling. It employs Euclidean loss instead of traditional cross-entropy loss for training. Compared to static images, videos often contain more information and Uddin et al. (30) introduced a two-stream deep spatiotemporal network to assess depression levels from video data. This framework employs the Inception-ResNet-v2 for spatial data and a volume local directional number descriptor for facial motion analysis, enhanced by convolutional neural network (CNN) processing. It also features a multilayer bidirectional long short-term memory (Bi-LSTM) with temporal median pooling to integrate spatial and temporal features effectively. Then, He et al. (31) introduced an end-to-end trainable system for depression detection, utilizing a 3D CNN with a spatiotemporal feature aggregation module. This system utilizes a 3D DEP-NetVLAD aggregation method to effectively identify depression. In 2023, Rajawat et al. (32) introduced a fusion fuzzy logic model combined with deep learning to identify depression through facial expressions from image and video files. Their model uses a fuzzy algorithm and unordered fuzzy rule initiation for depression recognition, transforming facial expressions into detectable indicators of depression.

Furthermore, many multimodal networks based on audio and video have also achieved excellent results in the field of depression detection. Niu et al. (33) introduced a novel Spatio-Temporal Attention network combined with a Multimodal Attention Feature Fusion strategy for predicting depression levels by capturing multimodal cues in 2020. In addition, Sun et al. (34) developed a multi-modal adaptive fusion transformer network and it is tailored to extract long-term context from uni-modal data. This network employs an adaptive fusion technique to integrate multimodal features effectively. Bucur et al. (35) introduced a unique time-enriched multimodal transformer architecture that leverages pre-trained models to extract image and text embeddings from social media posts in 2023. Operating at the user level, their model integrates time2vec positional embeddings to account for the timing of posts. Furthermore, they developed a variant designed to handle randomly sampled, unordered post sets, thereby increasing robustness against dataset noise. Li et al. (36) proposed a Decoupled Multimodal Distillation (DMD) framework to address modality heterogeneity in emotion recognition by separating each modality’s representation into modality-exclusive and modality-irrelevant components. A graph-based distillation unit (GD-Unit) enables dynamic, adaptive knowledge transfer between modalities via learned edge weights. This flexible structure improves feature discrimination and crossmodal alignment, achieving superior performance on standard multimodal emotion recognition benchmarks.

In summary, recent advancements in the field of automatic depression estimation have showcased the potential of deep learning and multimodal approaches in improving the accuracy and reliability of depression detection. Various methods utilizing speech signals, facial expressions, and video data have been developed, each contributing unique strengths and innovations. As the field continues to evolve, future research should focus on refining these models, addressing their limitations, and improve the effectiveness of depression detection.

## 3 Materials and methods

### 3.1 Dataset and preprocessing methods

This chapter provides an overview of the datasets utilized in this study, as well as the preprocessing methods applied to the raw data, encompassing audio, video, and text modalities.

#### 3.1.1 AVEC 2014 dataset

In this study, we utilize the Audio-Visual Emotion Recognition Challenge (AVEC) 2014 dataset (28). This dataset is among the few that offer unprocessed audio and video data, which are critical for analyzing nuanced behavioral cues and expressions associated with depression. The AVEC 2014 dataset includes 150 German participants (96 female and 54 male) and the subjects had a mean age of 31.5 years, with a standard deviation of 12.3 years, ranging from 18 to 63 years. Each of them completed two tasks to generate differentiated audiovisual data. The tasks selected are as follows: (1) Northwind dataset: Participants recite a passage from the fable “Die Sonne und der Wind” (The North Wind and the Sun) in German. (2) Freeform dataset: Participants express themselves spontaneously by answering various prompts, such as: “What is your favorite dish?”; “What was your best gift, and why?”; or “Discuss a sad childhood memory.” All responses are answered in German. Therefore, these tasks have been specifically selected to enable a comprehensive analysis of both verbal and nonverbal cues associated with depression. Additionally, each participant’s depression severity is quantified using the Beck Depression Inventory-II (BDI-II) (37) scores, which serve as labels for the dataset. According to standard interpretation guidelines, BDI-II scores can be categorized as follows: 0–13 indicates minimal depression, 14–19 mild depression, 20–28 moderate depression, and 29–63 severe depression.

#### 3.1.2 Video preprocessing

**Figure 2:**
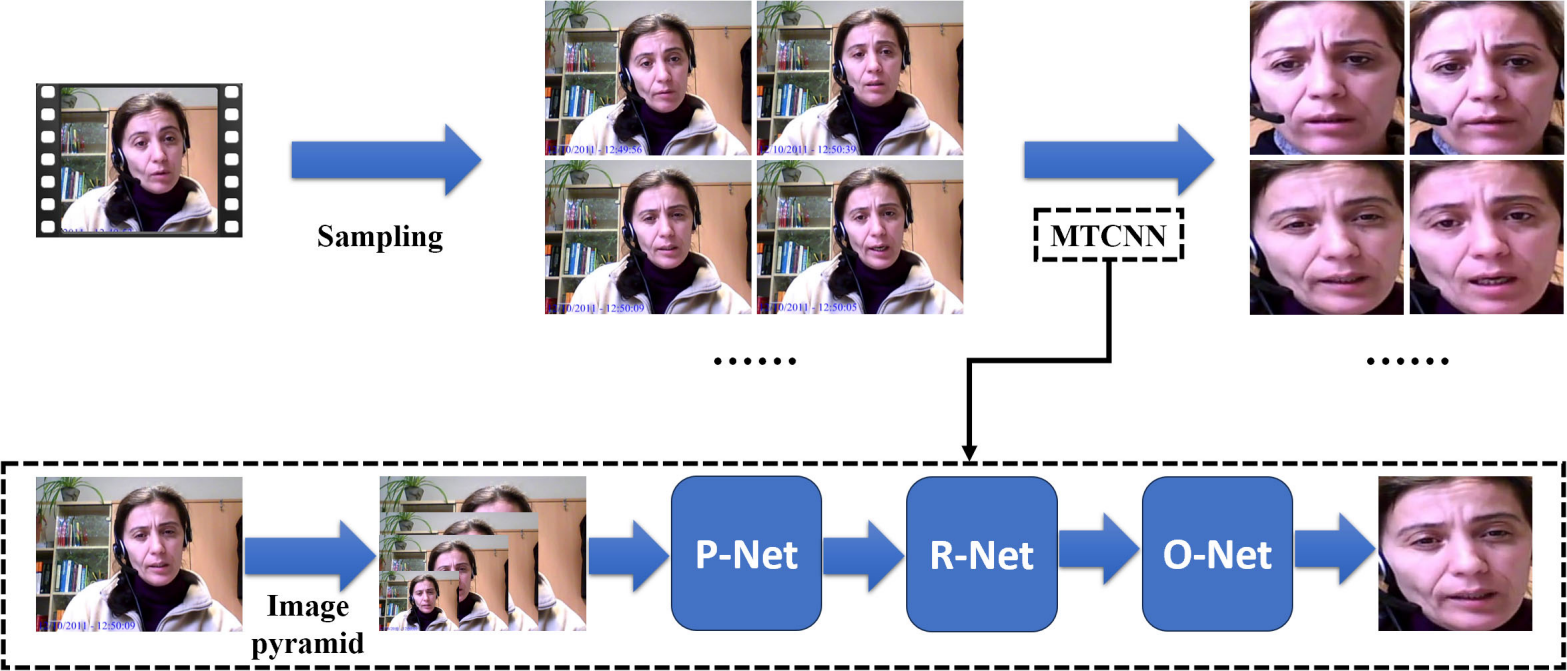
The preprocessing steps applied to the video data (MTCNN indicates Multi-task Cascaded Convolutional Neural Networks).

In this section, we detail the preprocessing steps applied to the video data of the AVEC 2014 dataset, and the process is depicted in **Figure 2**.

First, we perform sampling on the raw videos. Since the original videos vary in length, we employ an adaptive sampling interval approach. This method calculates the sampling interval based on the length of the video and the number of frames, ensuring that each video yields exactly one hundred frames after sampling.

To minimize the interference from the background and other irrelevant information, each frame is processed through Multi-task Cascaded Convolutional Neural Networks (MTCNN) (38). First, MTCNN employs an image pyramid (39) to handle various face sizes within the frame, allowing it to perform detection across multiple scales. Then, the processed images are sent to Proposal Network (P-Net). It generates candidate facial regions by rapidly scanning the resized images, proposing potential areas that likely contain faces. After that, the outputs of the P-Net are sent to the Refine Network (R-Net) and this stage refines these candidates, filtering out false positives. Finally, the outputs of R-Net are sent to the Output Network and this stage also provides final bounding boxes and associated confidence scores to confirm the presence of facial features and output the final processed frames.

Following this preprocessing procedure, we have successfully processed the video data for 150 participants, with each set consisting of 100 frames.

#### 3.1.3 Audio preprocessing

The processing of audio data in this study focuses on both global and local features, utilizing eGeMAPS (40) and MFCC (41) respectively. This section will detail the preprocessing steps used to extract these features, ensuring a comprehensive analysis for depression detection.

**Figure 3:**
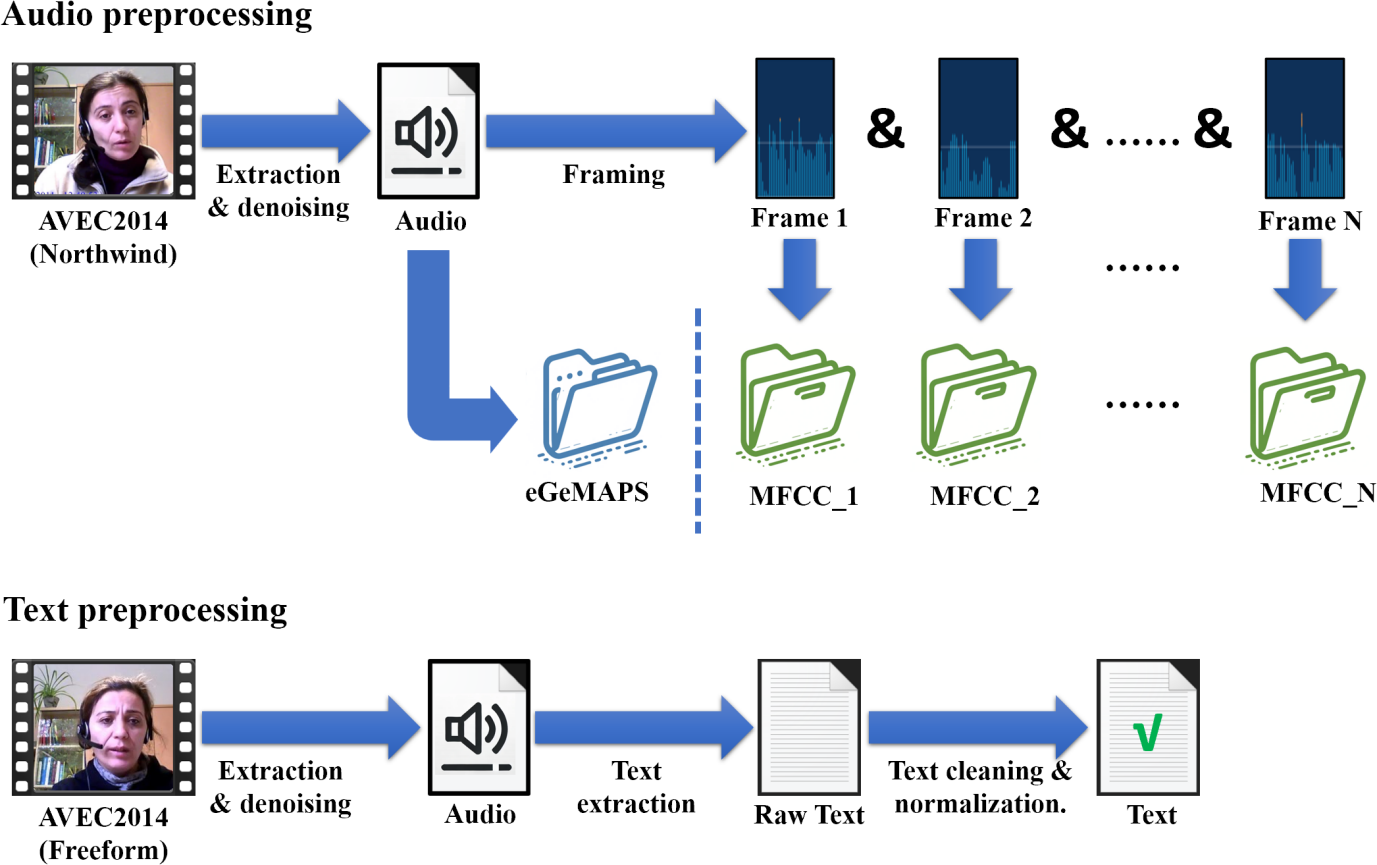
The preprocessing steps applied to the audio data and text data.

To control variables that might affect experimental outcomes, the extraction of audio features employed the Northwind dataset from AVEC2014. This ensures consistency in the content spoken by all participants. After extracting audio from original video files, the audio undergoes a noise reduction process. This involves spectral gating and wavelet denoising to remove noise while preserving essential aspects of the voice. Following the denoising, MFCC and eGeMAPS are extracted and the overall process is shown in the upper part of **Figure 3**.

MFCCs are widely used in speech and audio processing and they play a pivotal role in capturing the local features of audio for our analysis. MFCCs are derived from the Fourier transform of a signal, with a focus on the Mel scale, which approximates the human auditory system’s response more closely than the linearly-spaced frequency bands used in the standard cepstral analysis. The calculation of MFCCs is as Equation 1:


(1) $$f_{mel}=2595\log_{10}(1+\frac{f}{700})$$

Initially, each audio track is segmented into 1,000 frames and for each frame, we extract the first 20 MFCCs. To further enhance the descriptive power of our features, we compute both the first and second derivatives of these coefficients, effectively capturing the dynamic changes in the cepstral features over time. As a result, each frame is represented by 60 MFCC features (20 coefficients plus their first and second derivatives), culminating in a 1000 * 60 feature matrix for each audio sample. This feature set allows us to analyze the local characteristics of the speech.

Meanwhile, we utilize the eGeMAPS (40) to represent the global features of speech. Unlike the MFCCs which focus on capturing fine-grained local properties of audio, eGeMAPS is designed to encapsulate the overall statistical characteristics of speech. This feature set is particularly effective for its comprehensive coverage of voice attributes that are commonly implicated in emotional states and psychological conditions.

eGeMAPS is an acoustic parameter set built on the Geneva Minimalistic Acoustic Parameter Set (GeMAPS). eGeMAPS consists of 88 features derived from basic acoustic descriptors (Low-Level Descriptors, LLDs) through various statistical methods, and these statistical features are known as High-Level Statistics (HSFs). The features cover multiple acoustic aspects including frequency, energy, and spectral properties. Each audio sample in our dataset is processed to extract a complete set of these eGeMAPS features, ultimately transforming the entire speech signal into a single 1 * 88 vector. This representation captures the global attributes of the speech.

#### 3.1.4 Text preprocessing

To capture the diverse semantic information expressed by participants during interviews, our study begins by extracting textual data from the AVEC 2014 Freeform dataset, and the process is shown in the lower part of **Figure 3**. The first step involves extracting audio from the video recordings, followed by a noise reduction process to ensure the clarity of human voices. Several preprocessing techniques are employed here, including spectral gating to reduce background noise and dynamic range compression to maintain a consistent audio level.

Once the audio is cleaned, it is converted into German text. Following transcription, the German text undergoes text cleaning and normalization. This involves transforming all text to lowercase, stripping away punctuation and special characters, and discarding stopwords that do not add significant value to the depression detection.

Thus, we have thoroughly and meticulously preprocessed the data for 150 participants, obtaining refined final datasets across three modalities: audio, video, and text. This comprehensive preparation ensures data well-suited for the subsequent stages of our analysis.

### 3.2 Framework of IMDD-Net

**Figure 4:**
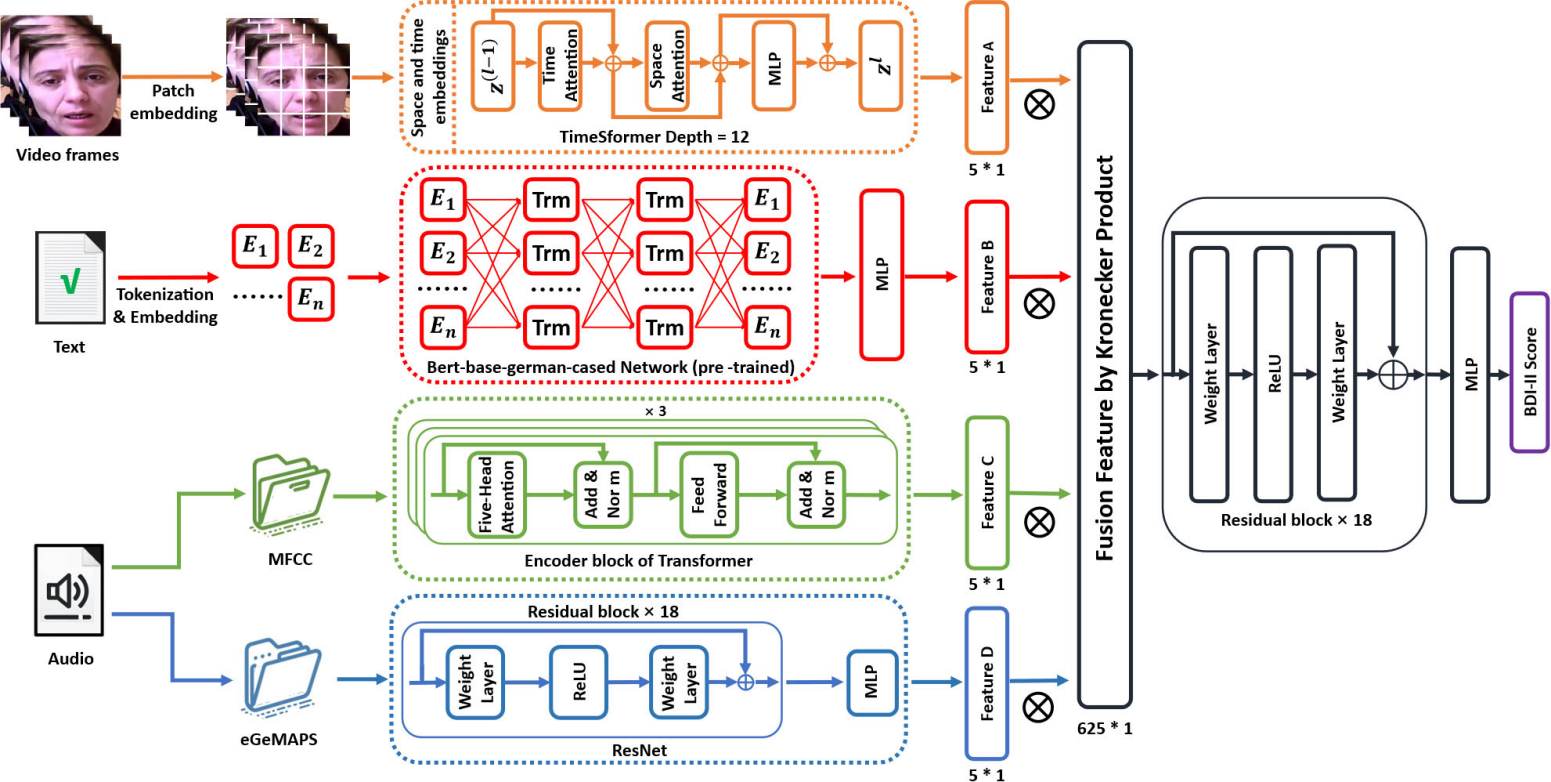
Framework of the proposed Integrative Multimodal Depression Detection Network (IMDD-Net).

This chapter is dedicated to detailing the architecture of the IMDD-Net, a multimodal network designed for assessing depression. The IMDD-Net integrates audio, text, and video modalities, using both local and global information within each modality to enhance estimation accuracy. The network processes these modalities through four specialized channels and a multimodal fusion and inference process, culminating in the output of BDI-II scores to evaluate the severity of depressive disorders. The architecture of the network is illustrated in **Figure 4**. In the following sections, the composition and functionalities of the IMDD-Net’s architecture will be introduced.

#### 3.2.1 Video feature extraction subnetwork

In order to capture both local and global features from video data effectively, the TimeSformer (42) network has been selected for the video modality channel of the IMDD-Net. The TimeSformer builds upon the Vision Transformer (ViT) (43) by integrating temporal processing capabilities, allowing it to effectively address the dynamic nature of video data. Specifically, the TimeSformer extends the Vision Transformer architecture by incorporating temporal dimensions into the self-attention mechanism. This enables the model to process sequences of video frames rather than static images.

**Figure 5:**
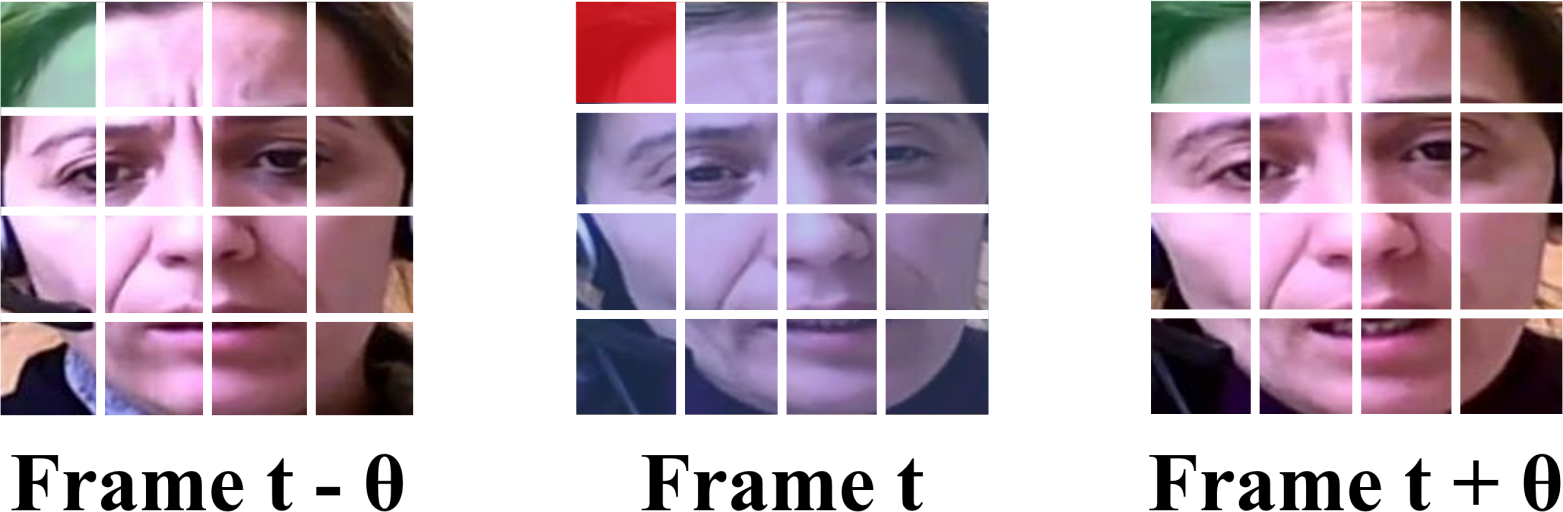
Local & global features extracted by space & time attention of TimeSformer.

Initially, each frame (64 * 64 pixels) of the video is divided into patches of size 8 * 8 pixels. These patches are then linearly embedded into 512-dimensional vectors. After patch embedding, each patch is concatenated with learned spatial (position within a frame) and temporal (position within the sequence) embeddings. Space and time embeddings enhance the model’s ability to interpret the positional context of each patch both within individual frames and across the sequence. The embedded patches are processed through multiple layers of spatial and temporal self-attention mechanisms. Each layer consists of 12 blocks (depth=12), where both local interactions within frames and global interactions across frames are captured (**Figure 5**). Each attention block is followed by a multilayer perceptron (MLP) and the output from the final layer of the TimeSformer is passed through a linear layer that shapes the output into a high-dimensional feature vector of size 5*1 per video. This vector represents the key features extracted and processed from the video which contain both local and global features.

#### 3.2.2 Textual feature extraction subnetwork

In the text modality channel of the IMDD-Net, we leverage the capabilities of the Bidirectional Encoder Representations from Transformers (BERT) (27) to analyze textual data. BERT is particularly well-suited for this task due to its ability to capture both local and global contextual information from text. Unlike directional models, which read the text input sequentially (left-to-right or right-to-left), the BERT model reads the entire sequence of words at once. This characteristic allows BERT to capture the meaning of a specific word in different contexts, providing a deeper understanding of the text as a whole.

For our study, we utilize the German variant of BERT, specifically the Bert-base-german-cased model, which has been pre-trained on a large corpus of German text. The model features an embedding layer that converts tokenized text into numerical vectors. Text data is initially processed using the BertTokenizer, and adjusts the length of these sentences to a uniform 350 tokens by padding. The subsequent transformer blocks process the input using self-attention mechanisms, followed by an MLP layer at the end to transform the token embeddings into a single vector.

The final output is a high-dimensional feature vector sized 5 * 1 for each piece of text, encapsulating both local and global contextual information.

#### 3.2.3 Audio feature extraction subnetwork

The audio modality channel in the IMDD-Net processes audio data to capture both local and global features.

Local Feature Extraction with MFCC: The audio files are first processed to extract MFCC, which focus on local information at the frame level. Each audio file is segmented into frames, and for each frame, 60 MFCC features are computed, resulting in a feature matrix of size 60 * 1000. These MFCC features are then processed through a Transformer model with 8 attention heads and a hidden dimension of 64. The Transformer encoder is composed of 3 layers and the 5 * 1 feature vector representing local speech information is outputted.

Global Feature Extraction with eGeMAPS: In parallel, eGeMAPS features are extracted to capture the global acoustic properties of the audio file and the size of input is 88 * 1. The eGeMAPS features are processed using a network structured similar to ResNet, which is adapted here for one-dimensional audio signal processing. This network is composed of 18 residual blocks and each block includes layers of linear transformations with skip connections. After processing through an MLP, the 5 * 1 high-dimensional feature vector representing global audio information is outputted.

At this point, we have obtained two vectors that respectively represent the global and local features of the audio information.

#### 3.2.4 Multimodal feature fusion and inference

In this final phase of the IMDD-Net, we converge the extracted features from different modalities to form a unified representation, leveraging the power of multimodal data fusion to enhance estimation precision.

The use of the Kronecker product in our fusion process allows for a detailed interaction between features from different modalities. Unlike simpler fusion techniques such as concatenation or averaging, the Kronecker product facilitates a richer and more expressive combination by mathematically intertwining the feature sets, thus capturing both inter-modal and intra-modal dependencies.

The Kronecker product, denoted by ⊗ , is a mathematical operation on two matrices. For matrices A of size m*n and B of size p*q , the Kronecker product A⊗B is a block matrix of size mp*nq . The operation is defined as Equation 2:


(2) $$A⊗B=[\begin{array}{ccc} a_{11}B & \ldots & a_{1n}B\\ \ldots & \ldots & \ldots \\ a_{m1}B & \ldots & a_{mn}B \end{array}]$$

where each element $a_{ij}$ of matrix A is multiplied by matrix B .

In the context of our IMDD-Net, the 5 * 1 feature vectors from each modality (representing both global and local information) undergo a transformation through the Kronecker product, resulting in a combined feature matrix. When these 5 * 1 vectors from the three modalities (audio, video, text) are subjected to the Kronecker product sequentially, the dimensionality of the resulting feature matrix expands to 625 * 1. This expansion not only increases the feature space but also preserves the unique characteristics of each modality.

The 625 * 1 feature vector is then processed through a deep residual network consisting of 18 layers and the output from the ResNet is a predictive value that correlates with the Beck Depression Inventory-II (BDI-II) scores.

In summary, the architecture of the IMDD-Net is designed for multimodal data. By integrating and processing local and global features from video, audio, and text inputs through advanced models, the system improves the accuracy of depression estimation and provides new insights for detecting depression.

### 3.3 Training methodology

The training and validation strategy of the IMDD-Net is designed to ensure a robust and comprehensive assessment of the model’s performance.

The dataset consists of data from 150 participants. To validate the effectiveness and stability of the model, five-fold cross-validation is employed. The dataset is divided into five equal parts randomly. In each fold, one part is held out as a validation set while the other four parts are used for training. This process is repeated five times, with each of the five parts used exactly once as the validation set.

The Huber loss function is selected because it is an error metric that combines the best aspects of L1 norm (mean absolute error) and L2 norm (mean squared error) loss functions, making it particularly effective for regression problems.

The Huber loss is defined by the Equation 3:


(3) $$L_{\delta }(y,\hat{y})=\{\begin{array}{c} \frac{1}{2}{\left(y-\hat{y}\right)}^{2},\;\;for\;|y-\hat{y}|\leq \delta \\ \delta (|y-\hat{y}|-\frac{1}{2}\delta ),\;\;for|y-\hat{y}|\;>\delta \end{array}$$

where y represents the true value, $\hat{y}$ is the predicted value and δ is a threshold parameter that defines the boundary between the quadratic loss and the linear loss. This threshold is adjusted as 1 ( δ=1 ) in our experiment.

The optimizer used is AdamW (Initial learning rate = 0.001), which combines the advantages of AdaGrad and RMSProp optimization methods and includes weight decay (weight_decay=0.01) regularization to prevent overfitting.

To evaluate the performance of our model, we adopt Mean Absolute Error (MAE) and Root Mean Square Error (RMSE) as primary evaluation metrics. These two measures are widely used in the field of automatic depression estimation, particularly on the AVEC 2014 dataset, allowing for direct and fair comparisons with prior state-of-the-art methods. MAE provides a straightforward interpretation of average error, while RMSE penalizes larger deviations more heavily, offering insight into prediction stability. MAE and RMSE which are defined as Equations 4 and 5:


(4) $$RMSE=\sqrt{\frac{1}{N}\sum {\left(r_{i}-r_{i}^{'}\right)}^{2}}$$

(5) $$MAE=\frac{1}{N}\sum \left|r_{i}-r_{i}^{'}\right|$$

where N is the total number of observations, $r_{i}$ is the prediction from the model, and $r_{i}^{'}$ is the actual observed value.

**Figure 6:**
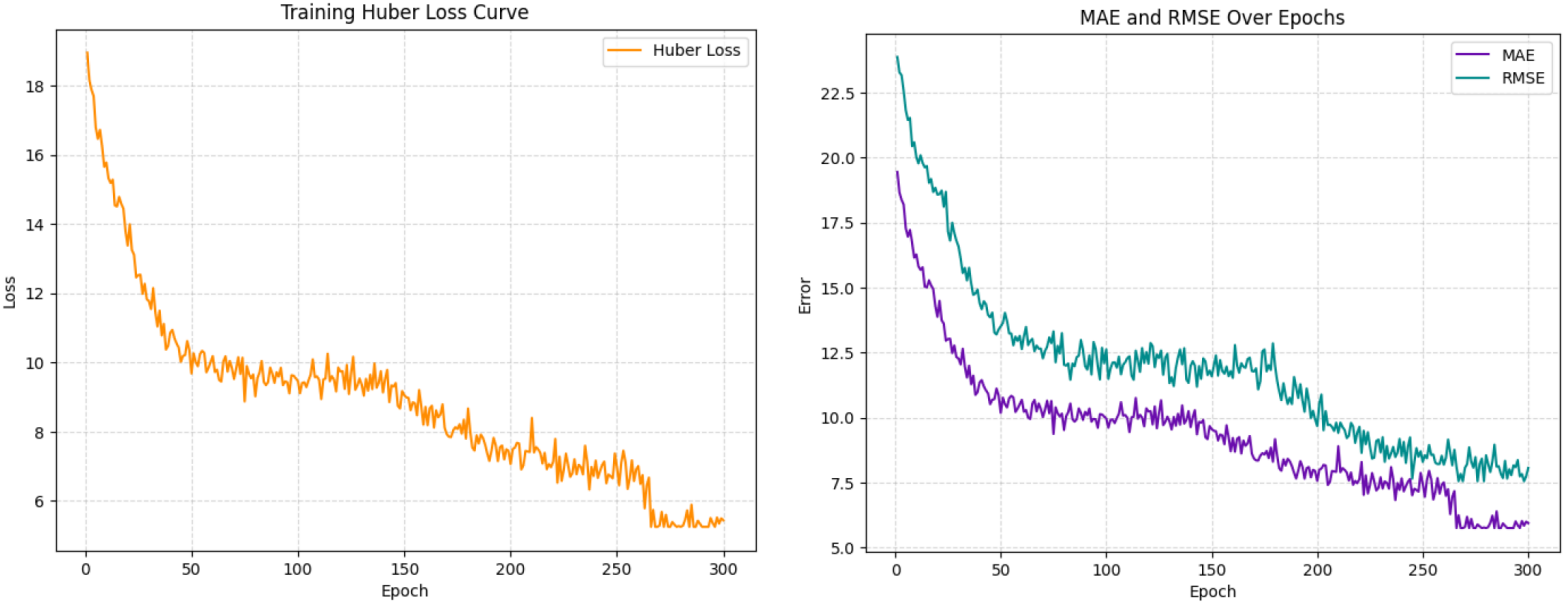
Averaged training curves of Huber loss, MAE, and RMSE over 300 epochs across five cross-validation folds.

The model was trained for 300 epochs, and **Figure 6** presents the Huber loss, MAE, and RMSE curves averaged across the five cross-validation folds.

## 4 Results

### 4.1 Experiments result of IMDD-Net

In this section, we present the experimental results of the IMDD-Net, comparing its performance against state-of-the-art (SOTA) methods and the baseline from the AVEC 2014 challenge. The evaluation metrics used to assess the performance are RMSE and MAE.

As shown in **Table 1**, our model has achieved state-of-the-art performance with an RMSE of 7.55 and an MAE of 5.75. The AVEC 2014 baseline has an RMSE of 9.89 and an MAE of 7.89 (28). Cai et al. (44) proposed an end-to-end time-domain channel attention network (TDCA-Net) for depression detection and on the AVEC 2014 dataset, their model achieved an RMSE of 8.90 and an MAE of 7.08. Additionally, Dong et al. (19) proposed a hierarchical depression detection model combining deep speaker recognition and speech emotion recognition features, achieving an RMSE of 8.73 and an MAE of 7.32 on the AVEC 2014 dataset. Moreover, Uddin et al. (30) developed a two-stream deep spatiotemporal network for depression level estimation, resulting in an RMSE of 8.78 and an MAE of 6.86. Furthermore, He et al. (31) introduced an end-to-end trainable intelligent system utilizing a 3D convolutional neural network with a spatiotemporal feature aggregation module achieving an RMSE of 8.42 and an MAE of 6.78 on the AVEC 2014 dataset and Shang et al. (45) proposed a method called Local Quaternion and Global Deep Network, which integrates local quaternion and global deep features for facial depression recognition, achieving an RMSE of 7.84 and an MAE of 6.08. Moreover, Melo et al. (46) introduced the Maximization and Differentiation Network to represent facial expression variations relevant for depression assessment, achieving an RMSE of 7.90 and an MAE of 6.19 on the AVEC 2014 dataset. Additionally, Niu et al. (47) proposed a multi-scale and multi-region facial dynamic representation method for depression prediction, achieving an RMSE of 7.98 and an MAE of 6.14 and Melo et al. (48) proposed a two-stream model with a novel temporal pooling method for capturing spatio-temporal dynamics in video clips, achieving an RMSE of 7.94 and an MAE of 6.20 on the AVEC 2014 dataset. Pan et al. (49) proposed the Spatial-Temporal Attention Depression Recognition Network, which enhances feature extraction by capturing global and local spatial-temporal information, achieving an RMSE of 7.75 and an MAE of 6.00 and Song et al. (50) proposed a method for video-based automatic depression analysis using multi-scale video-level features and novel spectral representations, achieving an RMSE of 7.15 and an MAE of 5.95 on the AVEC 2014 dataset.

**Table 1:** The comparison of different methods and their structures on the AVEC 2014 dataset.

Method	Network structure	Modality	RMSE**↓**	MAE↓
AVEC 2014 baseline (28)	Support Vector Regression	Audio-Video	9.89	7.89
(44)	An end-to-end time-domain channel attention network (TDCA-Net)	Audio	8.90	7.08
(19)	A hierarchical model combining deep speaker recognition and speech emotion recognition features	Audio	8.73	7.32
(30)	A two-stream deep spatiotemporal network	Video	8.78	6.96
(31)	An end-to-end trainable system utilizing a 3D convolutional neural network with a spatiotemporal feature aggregation module	Audio-Video	8.42	6.78
(45)	Local Quaternion and Global Deep Network	Video	7.84	6.08
(46)	Maximization and Differentiation Network	Video	7.90	6.19
(47)	A multi-scale and multi-region facial dynamic representation method	Video	7.98	6.14
(48)	A two-stream model with a novel temporal pooling method for capturing spatio-temporal dynamics in video clips	Video	7.94	6.20
(49)	Spatial-Temporal Attention Depression Recognition Network	Video	7.75	6.00
(50)	Using multi-scale video-level features and novel spectral representations	Video	**7.15**	5.95
**Ours**	Integrative Multimodal Depression Detection Network by using local and global multimodality features	Audio-Video-Text	7.55	**5.75**

The symbol "↓" indicates that lower values represent better performance (as for MSE and RMSE).

Bold values denote the best performance across methods.

In summary, our model has achieved state-of-the-art performance, demonstrating its effectiveness in depression detection. Notably, our model achieved the lowest MAE among all compared models, with a score of 5.75, indicating its superior accuracy in predicting depression severity. Furthermore, our model attained the second-lowest RMSE, with a score of 7.55, closely following the RMSE of 7.15 achieved by Song et al. [38]. These results underscore the robustness and precision of our IMDD-Net in integrating multimodal data and global and local features to enhance depression detection.

**Figure 7:**
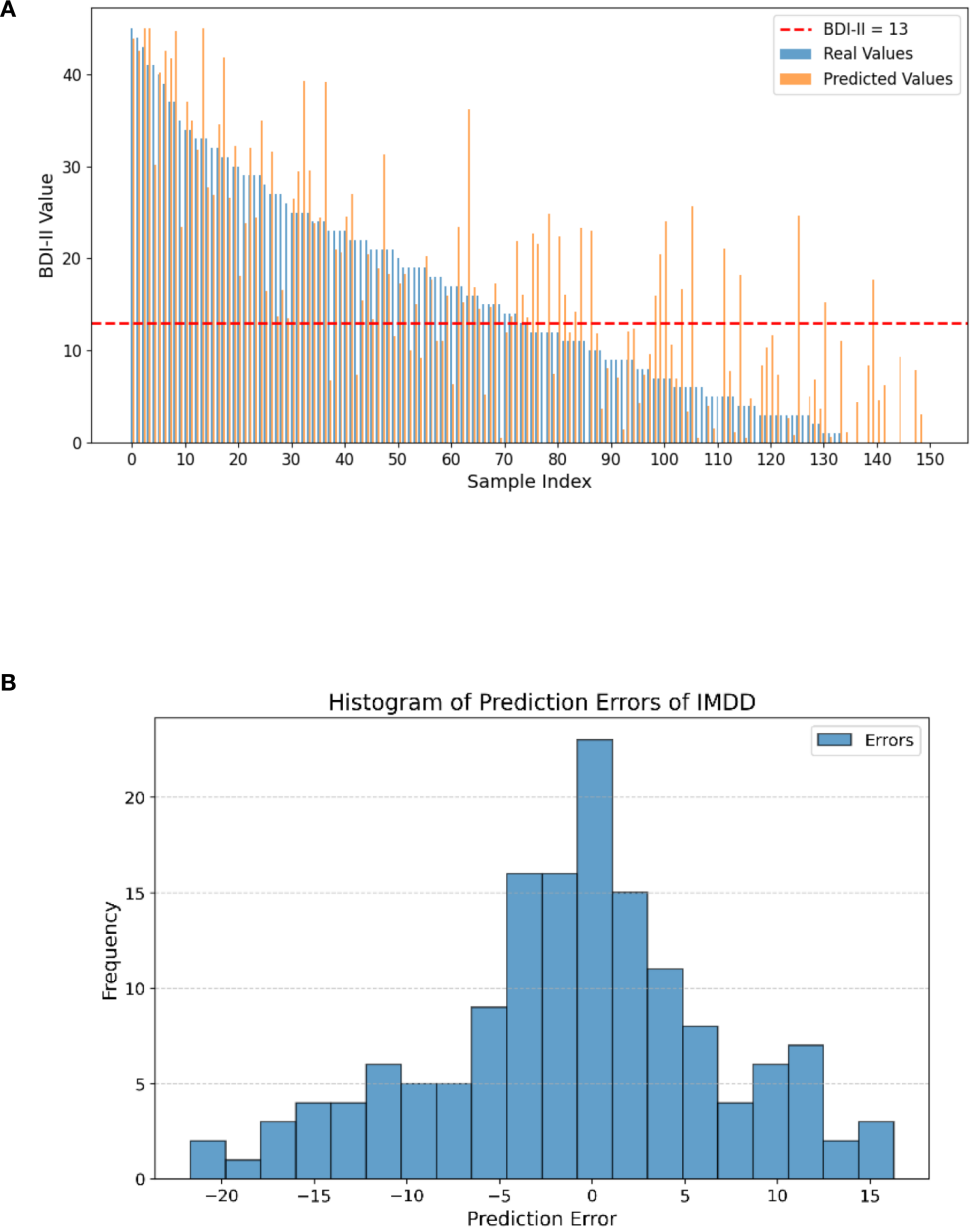
Differences between real and predicted BDI-II values and histogram of prediction errors of IMDD-Net. **(A)** The comparison of real and predicted BDI-II values; **(B)** Histogram of prediction errors.

In our study, we generated a BDI-II value comparison bar plot and an error histogram to evaluate the prediction performance of the IMDD-Net visually (**Figure 7**). **Figure 7A** presents a bar plot comparing the real and predicted BDI-II values for each sample. The x-axis corresponds to the sample indices, and the y-axis represents the BDI-II values. Each sample is represented by two bars: one for the real value and one for the predicted value. The red dashed line in the figure represents the threshold at a BDI-II score of 13. Typically, BDI-II scores below 13 are considered indicative of no depression, while scores above 13 suggest the presence of depressive symptoms. Analysis of **Figure 7A** reveals that the IMDD-Net tends to underestimate the actual BDI-II scores of individuals with depression (BDI-II scores greater than 13, (43 of 73 are underestimated), while overestimating the BDI-II scores of those without depression (BDI-II scores less than 13, 45 of 77 are overestimated).

As shown in **Figure 7B**, the error histogram illustrates the distribution of the prediction errors, which are calculated as the difference between the real and predicted BDI-II values. The histogram provides a visual representation of how closely the predicted values align with the actual values. The x-axis represents the prediction error, while the y-axis represents the frequency of each error value. The majority of the errors are concentrated around zero and 57.33% sample is located in the error range of ± 5, indicating that the IMDD-Net’s predictions are generally accurate.

**Figure 8:**
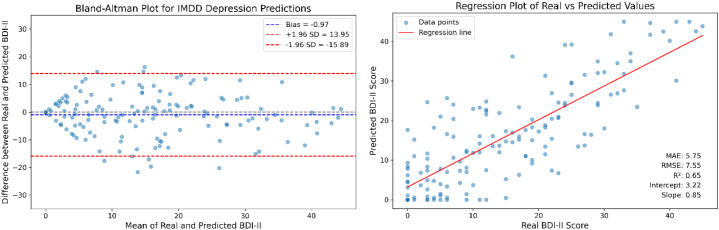
The Bland-Altman plot and regression analysis for IMDD-Net predictions.

To further illustrate the experimental results, we conducted a Brand-Altman analysis and performed a regression analysis. The Brand-Altman scatter plot and the regression plot are presented in **Figure 8** to illustrate the agreement and predictive accuracy of the IMDD-Net.

The Bland-Altman plot (the left part of **Figure 8**) compares the differences between the predicted BDI-II scores and the actual BDI-II scores against their averages. The mean difference (bias) is calculated to be −0.97 with a standard deviation of 7.61. The limits of agreement, defined as the mean difference ± 1.96 times the standard deviation, range from −15.89 to 13.95.

The regression analysis (the right part of **Figure 8**) shows the relationship between the predicted BDI-II scores and the actual BDI-II scores. The regression equation is given by: Predicted BDI-II = 0.85 × Actual BDI-II + 3.22. The coefficient of determination (R2) is calculated to be 0.65, indicating that approximately 65% of the variance in the actual BDI-II scores can be explained by the IMDD-Net’s predictions.

In summary, the Bland-Altman analysis shows good agreement between the predicted and actual BDI-II scores, while the regression analysis confirms the predictive accuracy of the IMDD-Net. These results collectively highlight the effectiveness of the IMDD-Net in assessing depression severity.

### 4.2 Classification performance analysis

**Figure 9:**
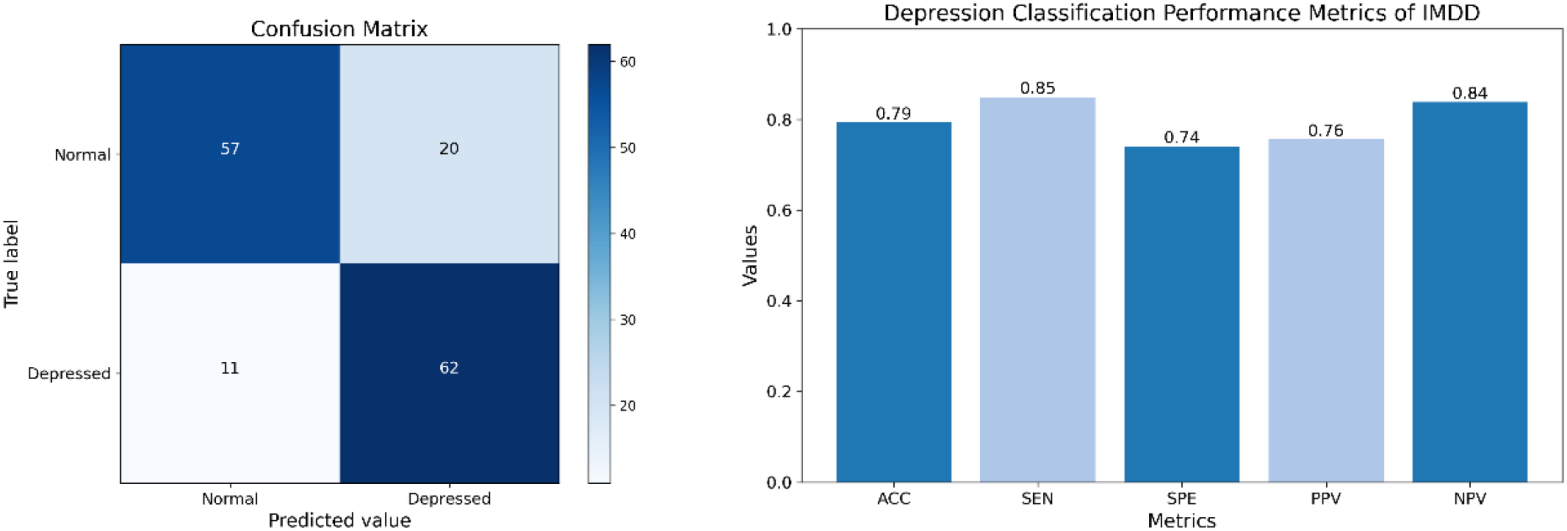
The confusion matrix and classification performance of IMDD-Net.

To further validate the effectiveness of IMDD-Net, we performed a classification analysis using a threshold of 13 on the BDI-II scores. Participants with scores above 13 were classified as depressed, while those with scores of 13 or below were classified as non-depressed. The confusion matrix (The left part of **Figure 9**) provides a detailed breakdown of the classification results, showcasing the true positive (TP) is 62, false positive (FP) is 20, true negative (TN) is 57, and false negative (FN) is 11. Therefore, the performance metrics are calculated as follows: Accuracy (ACC) is approximately 79.3%, Sensitivity (SEN) is 84.9%, Specificity (SPE) is 74.0%, Positive Predictive Value (PPV) is 75.6%, and Negative Predictive Value (NPV) is 83.8% (The right part of **Figure 9**).

### 4.3 Ablation experiment

To further emphasize the importance of multimodal data in the detection of depression, we conducted an ablation study. In this study, we evaluated the performance of the IMDD-Net using individual modalities and combinations of two modalities and compared the results with the complete multimodal configuration.

For the ablation study, we considered the following configurations: (a) Audio Only (MFCC and eGeMAPS); (b) Video Only; (c) Text Only; (d) Audio + Video; (e) Audio + Text; (f) Video + Text; (g) IMDD (Audio + Video + Text). Each configuration was used to train and test the model independently, following the same training methodology as described in the previous sections.

The performance of each configuration was evaluated by RMSE and MAE. The results are summarized in **Table 2**. Among the single modalities, the text modality achieves the best performance with the lowest RMSE (7.81) and MAE (6.36). For dual-modality combinations, the Video + Text configuration yields the best results with an RMSE of 7.66 and an MAE of 5.84. Previous clinical psychology studies have indicated that the relationship between language users (e.g., speakers or writers) and their texts is meaningful and shows considerable promise for the depression detection (51). A study (51) also suggests that it may be possible to identify individuals at risk for depression through text-based analysis, which aligns with the results of our ablation experiments.

**Table 2:** The result of the ablation experiment of IMDD-Net.

Model variant	RMSE↓	MAE↓
Audio Only	8.49	6.88
Video Only	8.27	6.63
Text Only	7.81	6.36
Audio + Video	7.72	6.05
Audio + Text	7.68	5.99
Video + Text	7.66	5.84
IMDD (Audio + Video + Text)	**7.55**	**5.75**

The symbol "↓" indicates that lower values represent better performance (as for MSE and RMSE).

Bold values denote the best performance across methods.

**Figure 10:**
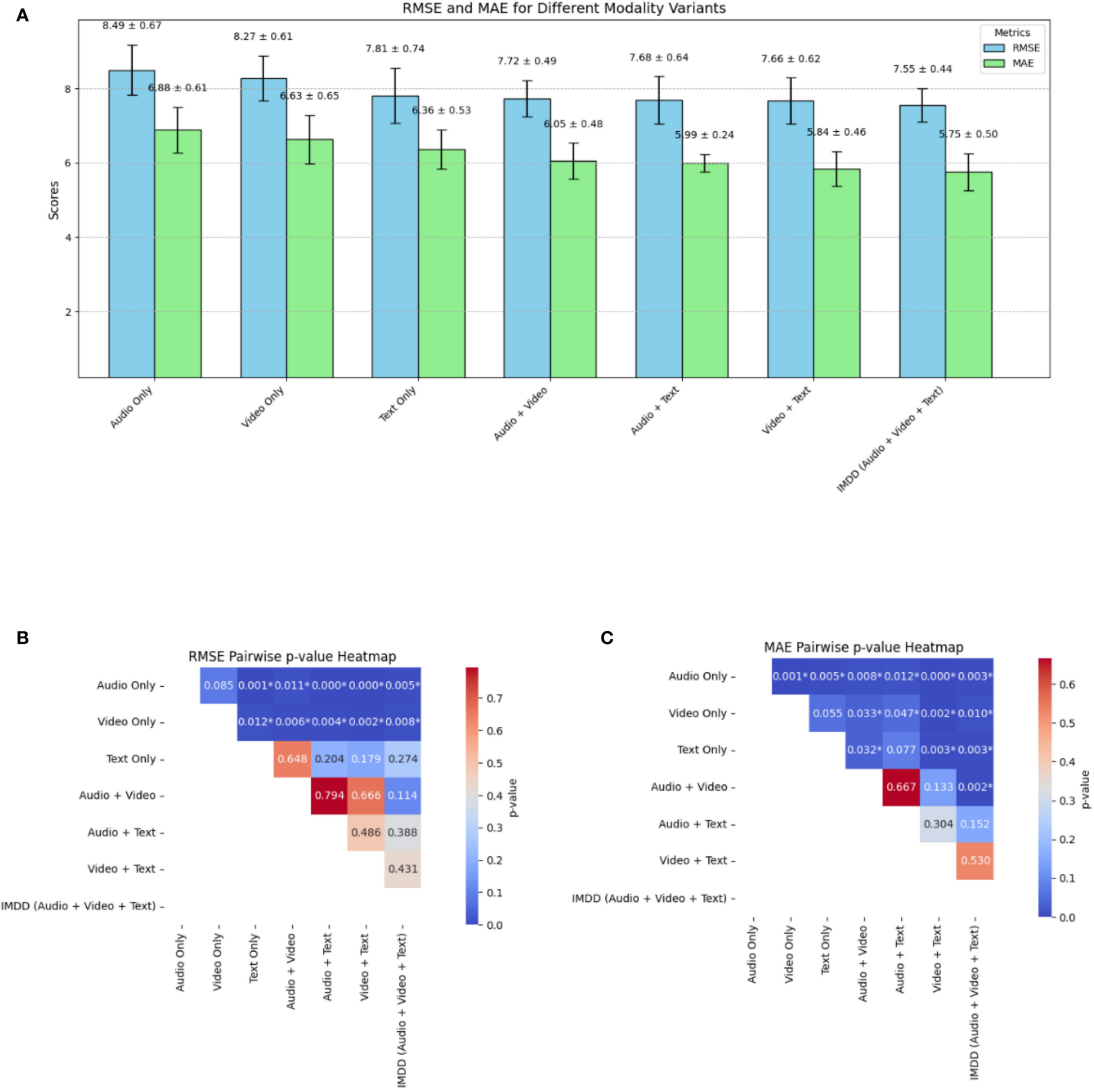
The result of the ablation experiment and statistical analysis. **(A)** RMSE and MAE. **(B)** Pairwise p-value heatmap of RMSE. **(C)** Pairwise p-value heatmap of MAE. (* indicates a significant difference.).

The ablation experiments and corresponding statistical analysis results are also shown in **Figure 10**, where the standard deviation is also included from five-fold cross-validation and paired t-tests are conducted on the RMSE and MAE metrics. The RMSE of the Audio Only and Video Only configuration is significantly higher than that of other configurations (p<0.05). The Text Only configuration presents significantly lower RMSE than other two single modality configurations (p<0.05), indicating that the information underlying the text might be more valuable for the depression detection or easier to be extracted by our IMDD-Net. The MAE of the IMDD (Audio + Video + Text) is significantly lower than that of single modality and Audio + Video configurations (p<0.05).

The ablation study clearly illustrates the critical role of multimodal data integration in the IMDD-Net. Each modality contributes unique and valuable information and enhances the network’s ability to detect depression accurately. The superior performance of the complete multimodal configuration underscores the necessity of leveraging diverse data sources in the field of depression detection.

An important experimental discovery in this study is the identification of the critical role played by multimodal data integration, specifically emphasizing the complementary nature of text, audio, and video modalities. Through ablation experiments, we observed that integrating text modality consistently improved performance more than audio or video alone, indicating that linguistic patterns provide highly discriminative signals for depression. This finding is consistent with clinical research, which suggests that linguistic expressions offer reliable and sensitive indicators of depressive states, capturing nuanced cognitive and emotional disturbances that might be less prominently reflected through facial expressions or vocal characteristics alone.

Furthermore, the comparative analysis of prediction errors and Bland-Altman agreement plots revealed specific behavioral patterns in our model’s predictions: the IMDD-Net tended to underestimate depressive severity in participants with higher BDI-II scores, possibly due to subtle or suppressed emotional cues in severely depressed individuals that are difficult to capture comprehensively from video or audio data alone. Conversely, it slightly overestimated scores in non-depressed individuals, suggesting a potential sensitivity toward ambiguous or transient emotional cues. These nuanced error patterns highlight opportunities for further refining modality-specific feature extraction and fusion techniques.

Overall, these experimental insights underscore the necessity of multimodal fusion in depression detection frameworks, particularly emphasizing the distinct contribution and sensitivity of linguistic information. This emphasizes the need for future research to prioritize advanced linguistic feature extraction methods and adaptive multimodal fusion strategies, ultimately improving the clinical utility and interpretability of automated depression detection systems.

## 5 Discussion

This study introduced IMDD-Net, a multimodal deep learning model designed to enhance depression detection by integrating video, audio, and text data. Our findings demonstrate that incorporating multiple modalities provides a more comprehensive and robust assessment of depressive symptoms. By leveraging both local and global feature extraction, IMDD-Net effectively captures short-term behavioral cues as well as long-term patterns in facial expressions, vocal tone, and linguistic structures. The model achieved an RMSE of 7.55 and an MAE of 5.75 for depression severity estimation, along with a classification accuracy of 79.3%, demonstrating high sensitivity (84.9%) and specificity (74.0%).

This study underscores the potential of multimodal deep learning in advancing objective, scalable, and accessible mental health assessments. By addressing current challenges, models like IMDD-Net could significantly contribute to the development of AI-driven tools for depression screening and monitoring, bridging the gap between computational psychiatry and clinical practice.

### 5.1 Effectiveness of multimodal data in depression prediction

The use of multimodal data, particularly the combination of audio, video, and text, has proven to be highly effective in depression prediction. Depression is a disorder that manifests through various behavioral, cognitive, and linguistic patterns (52). Studies have consistently shown that individuals with depression exhibit distinct facial expressions, speech patterns, and language use, which together provide a more comprehensive and reliable means of assessment (53, 54).

Facial expression analysis plays a crucial role in depression detection, as individuals suffering from depression often exhibit reduced facial variability, diminished smiling, and increased negative affect (55). Similarly, speech-based features such as prosody, pitch variation, articulation rate, and speech fluency offer valuable indicators of depressive states (56). Depressed individuals often demonstrate monotonous speech, slower articulation, increased hesitations, and longer response latencies (57), all of which can be extracted using acoustic analysis techniques such as MFCC and eGeMAPS. In addition to audio-visual cues, the linguistic patterns of individuals with depression often reflect negative sentiment, cognitive distortions, self-referential focus, and reduced syntactic complexity (51). By analyzing transcribed speech or written text, natural language processing (NLP) models can identify markers such as higher usage of first-person pronouns, excessive expressions of sadness or hopelessness, and reduced use of complex sentence structures (58).

While unimodal approaches relying on either facial expressions, speech, or text have demonstrated promising results, they often suffer from limited accuracy due to the heterogeneous nature of depressive symptoms (59). For instance, an individual may suppress facial expressions while still exhibiting changes in speech tone and linguistic patterns, or their speech may remain neutral while textual markers indicate distress. By integrating these three modalities, deep learning models can compensate for the weaknesses of each individual modality, leading to more robust and reliable depression prediction.

### 5.2 The role of global and local features in multimodal fusion

The integration of global and local features in multimodal learning plays a crucial role in capturing the complex manifestations of depression. Depression affects individuals in both momentary expressions and long-term behavioral patterns (60), making it essential for predictive models to analyze information across multiple temporal scales. Local features focus on fine-grained, short-term behavioral signals such as micro-expressions, brief tonal fluctuations in speech, and word-level linguistic markers, which provide immediate but transient indicators of depressive symptoms (61). On the other hand, global features capture long-term dependencies, trends in speech fluency, sustained emotional states in facial expressions, and discourse-level linguistic patterns, which reflect the broader psychological state of an individual over an extended period (62, 63).

Depression manifests at multiple temporal and behavioral levels, necessitating an approach that jointly models local variations and global trends. Future research should continue to explore adaptive multimodal fusion strategies that dynamically balance local and global information, ensuring that automated depression detection models remain both effective and clinically interpretable.

### 5.3 Limitations and future directions

In this paper, the IMDD-Net is introduced as a novel deep learning model designed to enhance the accuracy of depression detection by leveraging local and global features from video, audio, and text cues. This model effectively integrates multimodal data to provide a comprehensive analysis of depressive symptoms. By using the Kronecker product for multimodal fusion, our network explores deep interactions between modalities, further enhancing assessment accuracy. Experimental results demonstrate that the IMDD-Net achieves state-of-the-art performance. The robustness and precision of IMDD-Net are proved in identifying depression by extra experiment. In summary, via deep learning approach and video-audio-text multimodal data, IMDD-Net might be a useful tool of estimating depression risk in an undisturbed and convenient manner.

While the IMDD-Net has demonstrated state-of-the-art performance, there are several limitations that need further investigation. First, the IMDD-Net does not effectively address the issue of missing modalities. If any one of the input modalities (audio, video, or text) is absent, the model’s performance may be impacted. But it also means that depression can manifest through various modalities, encompassing diverse behavioral, emotional, and physiological indicators. Second, the integration of multimodal data and the use of advanced feature extraction techniques increase the computational complexity and resource requirements. This may hinder the deployment of the IMDD-Net in real-world, resource-constrained environments. Third, the current implementation of the IMDD-Net focuses on accuracy rather than real-time processing capabilities. For practical applications in clinical settings, the model needs to be optimized for faster inference without compromising accuracy. Furthermore, the current version of IMDD-Net has not been optimized for real-time deployment. Due to the model’s reliance on high-dimensional features, multiple deep architectures and Kronecker-based multimodal fusion, the computational load is relatively high. This may pose challenges for real-time inference, particularly in resource-constrained environments such as mobile platforms or telehealth applications. Finally, each modality (audio, video, text) comes with its own set of challenges, such as background noise in audio data, varying lighting conditions in video data, and the need for accurate transcription in text data. Addressing these issues comprehensively remains a challenge.

The ethical considerations are of crucial importance. While the IMDD-Net shows potential for use in clinical or diagnostic settings, its deployment raises important ethical considerations. First, the collection and analysis of audio, video, and textual data involve sensitive personal information, necessitating strict data privacy protection and informed consent protocols. Second, false positives may lead to unnecessary anxiety or stigmatization, while false negatives could result in missed opportunities for early intervention. Therefore, the model should not function as a standalone diagnostic tool but rather as an assistive system to support clinical decision-making. Additionally, bias in training data—such as demographic imbalance—could propagate disparities in predictions. Ensuring fairness, transparency, and human oversight will be essential in any future clinical deployment of the IMDD-Net.

Future work should focus on training and validating the IMDD-Net on larger and more diverse datasets, including data from various cultural backgrounds and different age groups. This would enhance the model’s generalizability and robustness. As a future direction, we also plan to collaborate with clinical partners to conduct prospective validation studies or pilot trials, aiming to assess the model’s reliability and generalizability in diverse patient populations. More efforts should be made to optimize the IMDD-Net for real-time processing through reducing computational complexity and improving the efficiency of the feature extraction and inference processes. Developing robust methods to handle missing modalities is crucial. This could involve creating imputation techniques or designing the model to be more resilient to incomplete input data, ensuring consistent performance regardless of data availability. Recovering missing modalities also represents a viable approach (64). By addressing these limitations and exploring these future directions, the IMDD-Net can provide a more reliable and efficient tool for the detection of depression.

## 6 Conclusion

In this study, we introduced the Integrative Multimodal Depression Detection Network (IMDD-Net), a novel deep-learning framework that effectively integrates video, audio, and textual modalities to enhance depression detection accuracy. By systematically capturing both local and global features within each modality and employing an advanced multimodal fusion strategy based on the Kronecker product, IMDD-Net provides a robust representation of depressive symptoms.

Experimental results conducted on the AVEC 2014 dataset demonstrate the superior performance of IMDD-Net, achieving state-of-the-art outcomes with a Root Mean Square Error (RMSE) of 7.55 and a Mean Absolute Error (MAE) of 5.75 in predicting BDI-II scores. The classification analysis further validates the model’s practical utility, yielding an accuracy of approximately 79.3% in distinguishing depressed individuals from non-depressed ones. Ablation studies underscore the critical contribution of each modality and reinforce the necessity of incorporating multimodal data.

Despite these promising outcomes, IMDD-Net faces several limitations that merit attention. The computational complexity associated with multimodal fusion and high-dimensional feature extraction poses challenges for real-time and resource-constrained applications. Moreover, the model currently lacks comprehensive clinical validation, limiting immediate deployment in clinical practice. Future studies will address these limitations by optimizing the computational efficiency, developing methods to handle incomplete modality inputs, and rigorously evaluating clinical validity through prospective trials.

Ultimately, by bridging advanced computational methods with multimodal behavioral indicators, IMDD-Net represents a significant step toward objective, accurate, and accessible depression screening, paving the way for enhanced mental health assessment practices.

## Data Availability

Publicly available datasets were analyzed in this study. This data can be found here: https://doi.org/10.1145/2647868.2647869.
